# Genetic Analysis Identifies *DDR2* as a Novel Gene Affecting Bone Mineral Density and Osteoporotic Fractures in Chinese Population

**DOI:** 10.1371/journal.pone.0117102

**Published:** 2015-02-06

**Authors:** Yan Guo, Tie-Lin Yang, Shan-Shan Dong, Han Yan, Ruo-Han Hao, Xiao-Feng Chen, Jia-Bin Chen, Qing Tian, Jian Li, Hui Shen, Hong-Wen Deng

**Affiliations:** 1 Key Laboratory of Biomedical Information Engineering of Ministry of Education, and Institute of Molecular Genetics, School of Life Science and Technology, Xi’an Jiaotong University, Xi’an, Shaanxi, P. R. China; 2 School of Public Health and Tropical Medicine, Tulane University, New Orleans, Louisiana, United States of America; Central China Normal University, CHINA

## Abstract

DDR2 gene, playing an essential role in regulating osteoblast differentiation and chondrocyte maturation, may influence bone mineral density (BMD) and osteoporosis, but the genetic variations actually leading to the association remain to be elucidated. Therefore, the aim of this study was to investigate whether the genetic variants in *DDR2* are associated with BMD and fracture risk. This study was performed in three samples from two ethnicities, including 1,300 Chinese Han subjects, 700 Chinese Han subjects (350 with osteoporotic hip fractures and 350 healthy controls) and 2,286 US white subjects. Twenty-eight SNPs in *DDR2* were genotyped and tested for associations with hip BMD and fractures. We identified 3 SNPs in *DDR2* significantly associated with hip BMD in the Chinese population after multiple testing adjustments, which were rs7521233 (*P* = 1.06×10^−4^, β: −0.018 for allele C), rs7553831 (*P* = 1.30×10^−4^, β: −0.018 for allele T), and rs6697469 (*P* = 1.59×10^−3^, β: −0.015 for allele C), separately. These three SNPs were in high linkage disequilibrium. Haplotype analyses detected two significantly associated haplotypes, including one haplotype in block 2 (*P* = 9.54×10^−4^, β: −0.016) where these three SNPs located. SNP rs6697469 was also associated with hip fractures (*P* = 0.043, OR: 1.42) in the Chinese population. The effect on fracture risk was consistent with its association with lower BMD. However, in the white population, we didn’t observe significant associations with hip BMD. eQTL analyses revealed that SNPs associated with BMD also affected *DDR2* mRNA expression levels in Chinese. Our findings, together with the prior biological evidence, suggest that *DDR2* could be a new candidate for osteoporosis in Chinese population. Our results also reveal an ethnic difference, which highlights the need for further genetic studies in each ethnic group.

## Introduction

Osteoporosis is a common disease characterized by low bone mineral density (BMD) and microarchitectural deterioration of bone tissue leading to increased risk of low-trauma osteoporotic fractures. Hip fractures are the most common and severe form of osteoporotic fractures associated with high mortality and tremendous health care costs [1]. The incidence of hip fractures continues to increase rapidly in developing countries [2]. One third of the world’s hip fractures now occur in Asia, mostly in China, and this rate will rise to 52% by 2050 [3]. Clinically, osteoporosis is defined through the measurement of BMD, which is the single best predictor of osteoporotic fractures [4,5].

Genetic factors have important influence on BMD and osteoporotic fractures, with heritability estimates of 0.6–0.8 for BMD [6] and ~0.5 for osteoporotic fractures [7]. However, the genetic variants actually involved remain largely unknown. In recent years, a number of genome-wide association studies (GWASs) on BMD have been successfully performed and more than 60 genome-wide significant loci have been reported [8]. Nevertheless, all the identified loci so far have modest effects on BMD and account for only a small proportion of the variance in liability to osteoporosis, suggesting that many more associated loci remain to be discovered.

*DDR2* gene (discoidin domain receptor 2) is located in chromosome 1q23.3 and encodes a member of the receptor tyrosine kinase (RTK) family that binds collagens and regulates cellular responses to the extracellular matrix [9]. DDR2 is demonstrated to be expressed in osteocytes, osteoblastic cells in subchondral bone, bone lining cells, tibia and cartilage [10,11,12]. In vivo studies provide functional evidence that DDR2 is required for normal bone development. Knockout of *DDR2* in mice leads to dwarfism, manifested by shortening of long bones [13,14]. In humans, mutations in *DDR2* gene are responsible for spondylometaepiphyseal dysplasia, which is manifested by short limbs with abnormal calcification [15]. Moreover, in vitro studies have shown that *DDR2* plays an essential role in osteoblast differentiation and chondrocyte maturation via a signaling pathway that involves MAP kinases and leads to the activation of the transcription factor RUNX2 [11,16]. Given the biological function of *DDR2* involved in bone, it is reasonable to hypothesize that *DDR2* may influence BMD and could be a new candidate gene for osteoporosis risk estimation, but the genetic variations actually leading to the association remain to be elucidated. Therefore, the aim of this study was to investigate the role of genetic variations of *DDR2* in BMD and fracture risk. Our study was performed in three samples from two ethnicities, including two Chinese Han populations and a US white population, in order to see whether the variants identified are common or ethnic-specific.

## Materials and Methods

### Subjects

The study was approved by the Research Administration of School of Life Science and Technology at Xi’an Jiaotong University and Institutional Review Board at Creighton University and University of Missouri-Kansas City. Signed informed consent documents were obtained from all study participants before entering the study. The basic characteristics of the study samples are summarized in Table 1, with additional descriptions below.

**Table 1:** Basic characteristics of the study subjects.

Trait	Chinese BMD sample	Chinese fracture sample		Caucasian sample
		Cases	Controls	
Number	1,300	350	350	2,286
Male/Female	600/700	124/226	173/177	558/1,727
Age (years)	33.42 (11.32)	69.35 (7.41)	69.54 (6.09)	51.37 (13.76)
Weight (kg)	59.63 (10.41)	59.15 (12.05)	59.61 (10.84)	75.27 (17.54)
Height (cm)	163.94 (8.11)	162.84 (8.31)	159.41 (9.20)	166.35 (8.47)
Hip BMD (g/cm^2^)	0.92 (0.13)	−	−	0.97 (0.18)

Note: Data are shown as mean (standard deviation, SD).

**Chinese samples**. We have two Chinese samples, one is BMD sample from a population-based cohort, and the other is fracture sample from a case-control cohort. All the subjects were unrelated northern Chinese Han adults living in the city of Xi’an and its neighboring areas. The BMD sample comprised 1,300 unrelated Chinese Han subjects. The inclusion and exclusion criteria have been detailed in our previous publication [17]. BMD (g/cm^2^) values at total hip were measured using Hologic 4500W machines (Hologic Inc., Bedford, MA, USA). The coefficient of variation (CV) value of the hip BMD was approximately 1.33%.

The fracture sample consisted of 350 cases with osteoporotic hip fractures and 350 elderly healthy controls. Hip fracture was diagnosed by orthopedic surgeons/radiologists according to radiological reports and X-rays. Healthy control subjects were selected from our established database as a ratio of 1:1 to cases and geography-matched. The inclusion and exclusion criteria for cases and controls have been described in detail in our earlier publication [17].

**Caucasian sample**. The Caucasian sample came from a population-based cohort, which consisted of 2,286 unrelated adults. All of the subjects were US Caucasians of Northern European origin living in Midwestern area. The sample description has been detailed in our previous study [18]. BMD values at hip were measured using the same model Hologic 4500W machines (Hologic Inc., Bedford, MA, USA) under the same strict protocols used in the Chinese sample. The CV value of the hip BMD was approximately 1.87%.

### SNP selection and genotyping

Genomic DNA was extracted from peripheral blood leukocytes using a commercial isolation kit (Gentra systems, Minneapolis, MN, USA) following the protocol of the kit. Selection of SNPs was done on data of the CHB population (Han Chinese in Beijing, China) provided by the HapMap project (Rel28 PhaseII + III, August10, on NCBI B36 assembly, dbSNP b126). We initially selected 30 SNPs in *DDR2* according to the following criteria: (i) minor allele frequency (MAF) > 0.05 in the CHB (Han Chinese in Beijing, China) population; (ii) an average density of 1 SNP per 5 kb; (iii) tagging SNP information.

For the Chinese BMD sample, SNP genotyping was carried out using MALDI-TOF mass spectrometry on a MassARRAY system (Sequenom, Inc., San Diego, CA) with iPLEX assay. Genotype calling was performed in real time with MassARRAY RT software version 3.0.0.4 and analyzed using the MassARRAY Typer software version 3.4 (Sequenom). Genotyping quality control procedures leading to SNP exclusion were call rate < 90%, MAF < 0.05 and *P* < 0.001 for deviations from Hardy–Weinberg equilibrium (HWE). The average call rate was 98% and the duplicate concordance rate was 99%. Of the 30 SNPs attempted, 28 were genotyped successfully. The basic characteristics of all the qualified SNPs are summarized in Table 2. For the Chinese fracture sample, SNP genotyping was performed using the Affymetrix Human Mapping 500K array set, which has been finished in our previous experiments [17]. For the Caucasian sample, SNP genotyping was performed using Genome-Wide Human SNP Array 6.0 (Affymetrix, Santa Clara, CA, USA), which has been detailed before [18]. For SNPs missing in the Affymetrix 500K or 6.0 arrays, we imputed genotypes using the IMPUTE program [19] to facilitate comparison of associations at the same SNPs. To guarantee the reliability of imputation, all imputed SNPs reached a calling threshold of 0.90, i.e., there was a 90% probability that an imputed genotype is true.

**Table 2:** Properties of all SNPs in *DDR2* tested in this study.

No.	SNP	Physical position	Genic position	Allele 1	Allele 2	MAF
1	rs4518873	162603729	Intron1	C	T	0.372
2	rs6671267	162608768	Intron1	G	A	0.095
3	rs10917577	162613975	Intron1	A	G	0.385
4	rs10799854	162619828	Intron1	C	T	0.113
5	rs3795641	162625020	Exon2	G	A	0.113
6	rs10799858	162630279	Intron2	G	A	0.175
7	rs12044481	162635875	Intron2	A	G	0.261
8	rs4657221	162642183	Intron2	G	A	0.168
9	rs7521233	162649471	Intron2	C	T	0.266
10	rs4657226	162653274	Intron2	G	A	0.388
11	rs6697469	162658773	Intron2	C	G	0.253
12	rs7553831	162661011	Intron2	T	G	0.264
13	rs10737487	162667331	Intron2	G	A	0.407
14	rs3923572	162671589	Intron2	T	C	0.237
15	rs10917588	162676191	Intron2	C	T	0.398
16	rs4559477	162681151	Intron2	T	G	0.365
17	rs16843872	162686362	Intron2	G	A	0.129
18	rs1039873	162692668	Intron3	C	G	0.226
19	rs2805034	162699597	Intron3	A	G	0.229
20	rs1510310	162703160	Intron3	C	G	0.232
21	rs2806423	162711989	Intron3	T	A	0.247
22	rs2684865	162721688	Intron3	T	C	0.222
23	rs2684866	162726281	Intron7	A	C	0.412
24	rs10799870	162733123	Intron9	T	C	0.173
25	rs2298258	162737116	Exon11	G	C	0.389
26	rs3738807	162741794	Intron12	T	C	0.227
27	rs1780007	162748025	Intron16	A	C	0.420
28	rs1779999	162753092	3’UTR	C	T	0.418

Note: MAF, minor allele frequency for allele A1 in the Chinese BMD sample.

### Statistical analyses

Before association analyses, to correct for potential population stratification that may result in spurious association results, we applied principal component analysis implemented in EIGENSTRAT [20] to all available genotypic data in the Chinese fracture and Caucasian samples, retaining the top ten principal components. For the BMD samples, significant parameters (*P* < 0.05) such as age, sex, height and weight were used as covariates to adjust for the raw BMD values. The distribution of the resulting residuals was tested for normality by Kolmogorov-Smirnov test. The residuals were used for association analyses. Linear regression implemented in PLINK [21] was fitted to examine association for each SNP assuming an additive inheritance model. Population haplotypes and their frequencies were inferred using Haploview software [22]. Haplotypes with estimated frequencies greater than 5% were included for association analyses by PLINK [21]. A raw *P* value of < 0.05 in our study was considered nominally significant, which was further subjected to Bonferroni correction to account for multiple comparisons. The significance threshold was set at a *P* value of less than 0.0018 for single SNP test (0.05/28 SNPs that were included in the association analyses) and 0.0038 for haplotype analysis (0.05/13 haplotypes that were included in the association analyses). For the validation analyses in the fracture sample, SNPTEST [19] was used to test for associations between genotypes and hip fractures. The covariates included age, sex, height, and weight.

### Expression quantitative trait locus (eQTL) analysis

We examined associations between SNPs and mRNA expression levels of the *DDR2* gene, to ascertain whether the SNPs associated with BMD affected expression of their transcript. Gene expression information was obtained from human lymphoblastoid cell lines (LCLs) of 210 unrelated individuals from HapMap populations in the NCBI Gene Expression Omnibus [23,24]. The sample included 60 white individuals from the CEU (Utah residents with northern and western European ancestry) and 45 Han Chinese individuals. SNP genotype data were derived from the corresponding HapMap Phase III dataset. The linear regression model implemented in PLINK [21] was used to determine associations between expression levels and genotypes.

## Results

### Association of SNPs in *DDR2* with BMD in the Chinese sample

**Fig 1:**
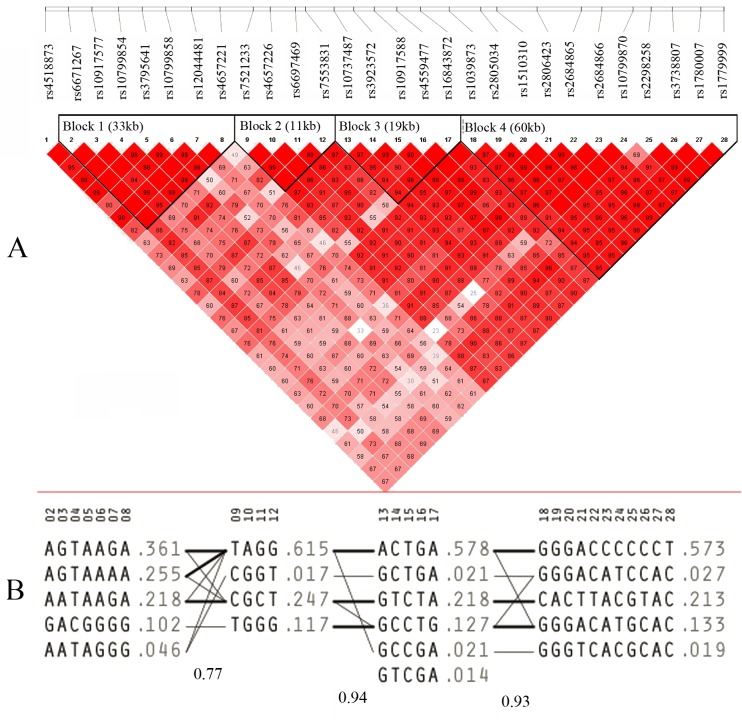
Linkage disequilibrium (LD) blocks and haplotype frequencies for *DDR2* in the Chinese BMD sample. (A). LD blocks are marked with triangles. Squares in red indicate strong LD. (B). Haplotypes in the four blocks across *DDR2*. The haplotype frequencies are shown to the right of each haplotype. The SNP numbers correspond to those given in Table 2.

The basic characteristics of the study subjects are provided in Table 1. The single SNP association results are summarized in Table 3. For the Chinese sample, there were 18 SNPs showing nominally significant associations with hip BMD (*P* < 0.05). After Bonferroni correction for multiple testing, 3 SNPs remained significant, which were rs7521233 (*P* = 1.06×10^−4^), rs7553831 (*P* = 1.30×10^−4^), and rs6697469 (*P* = 1.59×10^−3^), separately. Each minor allele of these 3 SNPs was associated with reduced hip BMD values with the effect size (β) of-0.018 (rs7521233-C), −0.018 (rs7553831-T), and-0.015 (rs6697469-C), respectively. All of these 3 SNPs are located at the intron 2 of *DDR2*. We further characterized the linkage disequilibrium (LD) block and haplotypes for *DDR2*. Four blocks in high LD were identified, which ranged in size from 11–60 kb (Fig. 1A). In these blocks, a total of 20 haplotypes were identified and 13 of them were with frequencies more than 0.05 (Fig. 1B). Table 4 listed the major association results for common haplotypes within each block. All of the 4 blocks showed nominally significant associations. After multiple testing adjustment, 2 haplotypes remained significant, which were haplotypes “CGCT” in block 2 (*P* = 9.54×10^−4^, β = −0.016) and “CACTTACGTAC” in block 4 (*P* = 2.63×10^−3^, β = −0.015). Block 2 included the above 3 significant SNPs (rs7521233, rs7553831, and rs6697469), presenting the consistent results between single SNP and haplotype analyses. As shown in Fig. 1, these 3 SNPs were in high LD with each other (pairwise LD *r* ^2^ and D’ > 0.9).

**Table 3:** Nominally significant association results for SNPs in *DDR2* with BMD and fractures.

No.^a^	SNP	Physical position	Genic position	A1/A2^b^	Chinese BMD sample			Chinese fracture sample			Caucasian sample			*P*_eQTL_ ^c^
					MAF	β	*P* value	MAF Cases	MAF Controls	*P* value	MAF	β	*P* value	
**9**	**rs7521233**	162649471	Intron2	C/T	0.266	−0.018	**1.06×10^−4^**	−	−	−	0.233	0.002	0.307	−
10	rs4657226	162653274	Intron2	G/A	0.388	−0.011	0.015	0.399	0.395	0.337	0.259	0.001	0.502	0.043
**11**	**rs6697469**	162658773	Intron2	C/G	0.253	−0.015	**1.59×10^−3^**	0.266	0.245	0.043	0.092	0.005	0.169	0.896
**12**	**rs7553831**	162661011	Intron2	T/G	0.264	−0.018	**1.30×10^−4^**	0.273	0.264	0.058	0.229	0.003	0.251	0.322
13	rs10737487	162667331	Intron2	G/A	0.407	−0.012	6.86×10^−3^	0.429	0.403	0.153	0.259	0.002	0.436	0.016
14	rs3923572	162671589	Intron2	T/C	0.237	−0.015	2.27×10^−3^	0.251	0.234	0.192	0.069	0.003	0.107	0.918
15	rs10917588	162676191	Intron2	C/T	0.398	−0.011	9.31×10^−3^	0.420	0.384	0.050	0.096	0.003	0.328	0.277
16	rs4559477	162681151	Intron2	T/G	0.365	−0.009	0.032	0.378	0.362	0.205	0.105	0.003	0.484	0.392
18	rs1039873	162692668	Intron3	C/G	0.226	−0.014	5.72×10^−3^	0.241	0.226	0.217	0.080	0.005	0.209	0.889
19	rs2805034	162699597	Intron3	A/G	0.229	−0.013	9.91×10^−3^	−	−	−	0.085	0.006	0.113	−
20	rs1510310	162703160	Intron3	C/G	0.232	−0.012	0.013	0.237	0.218	0.152	0.081	0.005	0.183	0.931
21	rs2806423	162711989	Intron3	T/A	0.247	−0.012	0.015	0.263	0.233	0.120	0.089	−0.003	0.416	0.931
22	rs2684865	162721688	Intron3	T/C	0.222	−0.012	0.014	−	−	−	0.080	0.005	0.183	−
23	rs2684866	162726281	Intron7	A/C	0.412	−0.009	0.037	0.402	0.397	0.593	0.225	0.001	0.655	0.030
25	rs2298258	162737116	Exon11	G/C	0.389	−0.009	0.036	−	−	−	0.146	−0.001	0.627	0.949
26	rs3738807	162741794	Intron12	T/C	0.227	−0.015	3.40×10^−3^	0.251	0.227	0.144	0.052	−0.007	0.139	0.921
27	rs1780007	162748025	Intron16	A/C	0.420	−0.010	0.020	0.427	0.427	0.846	0.206	0.001	0.751	0.416
28	rs1779999	162753092	3’UTR	C/T	0.418	−0.011	0.013	−	−	−	−	−	−	0.614

Note: MAF, minor allele frequency; OR, odds ratio; CI, confidence interval. “−“means the *P* value was not available for this SNP.

Significant *P* values after multiple testing adjustment (*P* < 0.0018) are shown in bold.

^a^ The IDs of SNPs correspond to Table 2.

^b^A1 represents the minor allele.

^c^ *P*_eQTL_ is shown for 45 HapMap Chinese Han individuals.

**Table 4:** Haplotype analysis in the Chinese BMD sample.

Block	SNPs^a^	Haplotype^b^	Frequency	*P*-value	β
Block 1	2–8	AGTAAGA	0.361	0.164	0.006
		AGTAAAA	0.255	0.667	0.002
		AATAAGA	0.218	4.59×10^−3^	−0.014
		GACGGGG	0.102	0.076	0.012
Block 2	9–12	TAGG	0.615	0.014	0.011
		**CGCT**	0.247	**9.54×10^−4^**	−0.016
		TGGG	0.117	0.124	0.010
Block 3	13–17	ACTGA	0.578	4.22×10^−3^	0.012
		GTCTA	0.218	6.12×10^−3^	−0.014
		GCCTG	0.127	0.617	0.003
Block 4	18–28	GGGACCCCCCT	0.573	0.021	0.010
		**CACTTACGTAC**	0.213	**2.63×10^−3^**	−0.015
		GGGACATGCAC	0.133	0.489	0.004

^a^ The IDs of SNPs correspond to Table 2.

^b^ For each block, we only listed the results of haplotypes with estimated frequencies more than 0.05.

Significant *P* values after multiple testing adjustment (*P* < 0.0038) are shown in bold.

### Association of the BMD associated SNPs in *DDR2* with fracture in the Chinese sample

We also examined associations between fracture and the above 18 SNPs associated with BMD (Table 3). SNP rs6697469 was found to be associated with hip fractures (*P* = 0.043), and the odds ratio (OR) was estimated to be 1.42 (95% confidence interval (CI): 1.04–1.95). Subjects with the heterozygote genotype CG had an increased risk of fracture, in comparison with those bearing GG genotype. Besides rs6697469, there were 2 additional SNPs showed marginally significant association signals with hip fractures, which were rs10917588 (*P* = 0.050) and rs7553831 (*P* = 0.058). These 2 SNPs also associated with increased risk of fractures, and the ORs were estimated to be 1.42 (95% CI: 1.02–1.97, genotypes CT versus TT at rs10917588), and 1.33 (95% CI: 0.97–1.81, genotypes TG versus GG at rs7553831), respectively. The effects on fracture risk for these 3 SNPs were totally consistent with their associations with lower BMD values.

### Replication in the white samples

We further checked the association results for the above 18 SNPs in our Caucasian sample (Table 3). However, we didn’t observe significant association signals between the above SNPs and hip BMD.

### eQTL analysis results

To investigate the functional relevance of the identified SNPs associated with BMD in *DDR2*, we performed eQTL analysis in Chinese and white samples from HapMap data, respectively. Three SNPs were significantly associated with *DDR2* mRNA expression levels in LCLs of 45 Chinese individuals (Table 3). However, no significant association was found in 60 white individuals (data not shown).

## Discussion

Previous studies have established the important role of *DDR2* in regulating osteoblast differentiation and chondrocyte maturation [11,16]. Deletions of *DDR2* in mice and humans have been found to be associated with dwarfism and short limbs [13,14,15]. Motivated by the functional role of *DDR2* involved in bone, we first performed an association study to investigate whether the common variants in *DDR2* influence BMD and fracture risk. We identified a cluster of SNPs in *DDR2* significantly associated with hip BMD and fractures in Chinese populations. The effects of *DDR2* on BMD and fracture were totally consistent. Taking into account of those biological evidence and our statistical findings, we suggest that *DDR2* might have potential role in BMD regulation and fracture pathogenesis.

To further evaluate the functional role of the identified SNPs in *DDR2*, we exploited eQTL analysis in human LCLs. Generally, analysis of *cis*-eQTL in tissue type relevant to phenotype is more informative than that in unrelated tissues. However, disease-related human tissues with large sample size are usually difficult to obtain for research purposes. Recent studies have shown that *cis*-eQTLs are conserved across tissues, and that tissue specific genetic variations comprise only a small proportion of gene expression diversity [25,26,27]. As for bone tissue, there is a large overlap in the transcriptomic effects of genetic variation between human osteoblasts and LCLs [28,29], suggesting that if genes are expressed across tissues, their allele-specific expression can be preserved and highly correlated across tissues. Therefore, we examined *cis*-eQTL associations to ascertain whether the SNPs associated with BMD affected expression of *DDR2* in human LCLs using public HapMap data. We found that the SNPs associated with BMD also affected the expression of *DDR2* in the Chinese individuals, but not in the white individuals, suggesting that the eQTLs we identified might be ethnic specific. Additionally, although the top 3 significant SNPs associated with BMD were not the significant eQTLs, another SNP, rs4657226, which was in high LD with these 3 SNPs and belonged to one block, was significantly associated with *DDR2* expression levels. The sample size of HapMap data is relatively small. Limited power could influence the detection of additional associations. Nevertheless, our results confirm the relevance of the association data and provide supplementary insights that are indirectly informative for osteoporosis.

Most of BMD associated loci are reported in white populations. Genes predisposing to the risk of osteoporosis may vary between white and Asian populations [30]. Our study found significant association for *DDR2* with hip BMD and fractures in Chinese populations. However, the significant associations were not replicated in our Caucasian population. Such results implied an ethnic differentiation. The minor allele of SNP rs6697469 was much common in Chinese than Caucasians, which may contribute to the overall effect and in part explain the ethnic difference. Conversely rs7521233 and rs7553831 showed similar MAF in both ethnic groups. The LD patterns for these 3 SNPs were differ between Chinese (pairwise LD *r* ^2^ > 0.9) and Caucasian (*r* ^2^ < 0.6) samples, which could also explain the different results partly. Nevertheless, our findings support that it is interesting and valuable to conduct genetic susceptibility studies in Asian populations, which could expand our knowledge of the genetic basis of osteoporosis.

We estimated the statistical power of our study using the Genetic Power Calculator program (http://pngu.mgh.harvard.edu/~purcell/gpc/qtlassoc.html). The conservative significance level was set at *P* = 0.0018. Assuming that a marker has a MAF of 0.05 and is in strong LD (*D’* = 0.98) with a functional variant that accounts for 1.0% variation of a phenotype, our Chinese BMD sample can achieve > 65% statistical power. For the 3 significant SNPs we identified, the proportion of BMD variation explained by each SNP was 1.15% (rs7521233), 1.15% (rs7553831), and 0.81% (rs6697469), respectively. We acknowledge that our study is not powerful to detect genetic variants with low effect size.

In summary, our study provides novel evidence that *DDR2* is associated with hip BMD and fractures in Chinese populations. Considering the biological function of *DDR2*, we suggest that *DDR2* could be a new candidate in determining the risk of osteoporosis and fractures. Our results also reveal some extent of ethnic difference for *DDR2*, which highlight the need for further genetic studies in each ethnic group. Our findings further advance our understanding of the genetic architecture of osteoporosis. Whether DDR2 could be exploited as a novel therapeutic agent for osteoporosis warrants further investigation.
